# Development of a mechanical adaptable, moisture retention capable, injectable and adhesive organohydrogel for nucleus pulposus repairing

**DOI:** 10.1093/rb/rbaf047

**Published:** 2025-05-19

**Authors:** Yaping Wang, Dong Wang, Chu Gao, Chuxin Zhou, Xiao Lin, Di Wang, Liu Yang, Huan Zhou, Lei Yang

**Affiliations:** Center for Health Science and Engineering, Hebei Key Laboratory of Biomaterials and Smart Theranostics, School of Health Sciences and Biomedical Engineering, Hebei University of Technology, Tianjin 300131, People’s Republic of China; Institute of Orthopedic Surgery, Xijing Hospital, Fourth Military Medical University, Xi'an 710032, Peoples’ Republic of China; Institute of Orthopedic Surgery, Xijing Hospital, Fourth Military Medical University, Xi'an 710032, Peoples’ Republic of China; Medical Research Institute, Northwestern Polytechnical University, Xi'an 710072, Peoples’ Republic of China; Institute of Orthopedic Surgery, Xijing Hospital, Fourth Military Medical University, Xi'an 710032, Peoples’ Republic of China; Orthopedic Institute and Department of Orthopedics, The First Affiliated Hospital, Soochow University, Suzhou 215000, People’s Republic of China; Institute of Orthopedic Surgery, Xijing Hospital, Fourth Military Medical University, Xi'an 710032, Peoples’ Republic of China; Institute of Orthopedic Surgery, Xijing Hospital, Fourth Military Medical University, Xi'an 710032, Peoples’ Republic of China; Medical Research Institute, Northwestern Polytechnical University, Xi'an 710072, Peoples’ Republic of China; Center for Health Science and Engineering, Hebei Key Laboratory of Biomaterials and Smart Theranostics, School of Health Sciences and Biomedical Engineering, Hebei University of Technology, Tianjin 300131, People’s Republic of China; Center for Health Science and Engineering, Hebei Key Laboratory of Biomaterials and Smart Theranostics, School of Health Sciences and Biomedical Engineering, Hebei University of Technology, Tianjin 300131, People’s Republic of China

**Keywords:** organohydrogel, nucleus pulposus, mechanical adaptable, intervertebral disc degeneration, reactive oxygen species

## Abstract

Developing mechanical adaptable injectable gel with nucleus pulposus (NP) repairing capability for minimally invasive treatment of intervertebral disc degeneration (IDD) is of great importance in medical practice. In current work, inspired by the outcomes of polyvinyl alcohol and glycerol based injectable organohydrogel (GPG) in IDD control and the great potential of animal glue in tissue adhesion, a novel injectable and self-crosslinking adhesive organohydrogel GPG-AG was fabricated. The mechanical performance of the GPG-AG was systematically studied, possessing viscoelastic properties close to NP accompanied with strong adhesion to intervertebral disc to avoid dynamic loading induced leakage postinjection. In addition, the swelling behavior, water retention capability and degradation of the organohydrogel *in situ* was also explored. *In vitro* cellular test showed the as-fabricated organohydrogel was able to upgrade aggrecan expression while downregulate matrix metallopeptidase-13 (MMP-13) synthesis. Astoundingly, the organohydrogel revealed anti-inflammation potential of alleviating excessive reactive oxygen species, consequently creating a favored microenvironment for NP repairing. The corresponding *in vivo* study showed the outcome in intervertebral disc height index of the GPG-AG treated group after needle puncture was superior to previously reported GPG and control group. Taken together, this organohydrogel is expected to serve as a promising candidate for IDD control.

## Graphical Abstract

**Figure:**
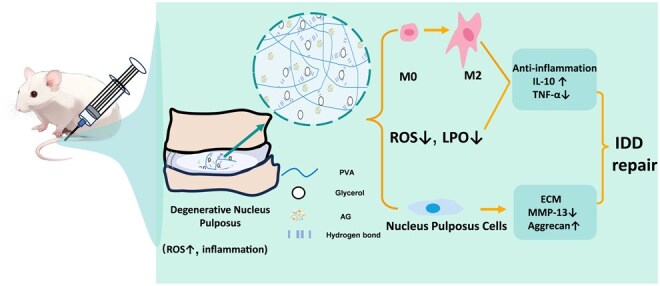

## Introduction

Low back pain (LBP) stands as one of the primary causes of global disability [1, 2], characterized by discomfort located between the lower rib margin and the buttocks [3–5]. With progressed understanding of LBP, intervertebral disc (IVD) degeneration has emerged as a pivotal contributing factor leading to this disease, along with increasing prevalence as global population aging acceleration [6, 7]. Physiologically, the IVD comprises a central, hydrated NP, surrounded by the lamellar annulus fibrosus (AF) and adjacent to the vertebral cartilage endplate (CEP). Among these physiological components, NP plays a vital role in supporting and transmitting spinal movement stress, cushioning external forces, maintaining disc shape and restricting vertebral motion range [8]. NP degeneration, driven by aging and chronic overload in daily life, is recognized as a key instigator of IDD, accompanied by a progressive loss of proteoglycan, type II collagen and resident cells. These alterations lead to adverse changes in the NP's water content and shock-absorbing capacity, ultimately compromising AF integrity and resulting in symptomatic LBP through abnormal and repetitive mechanical loading [9, 10].

Control of IDD by mitigating NP degradation poses a significant challenge in medical practice. Various strategies, including pharmacotherapy and physical rehabilitation therapy, have been applied for this purpose [11, 12]. However, long-term clinical trials of these attempts have demonstrated only limited and transient efficacy in pain relief [13]. When these conservative therapies fail to yield satisfactory outcomes, surgical interventions, such as disc replacement or spinal fusion, may be considered [14–16]. However, according to long-term follow-up results, new clinical symptoms were observed after surgery [17, 18]. In spite of that, the success rate of these interventions in restoring the local microenvironment, alleviating IDD and providing lasting relief from LBP remains suboptimal.

As IDD initially manifests in NP tissue, this physiological event thereby inspires worldwide efforts to restore the function and structural integrity of NP tissue [19]. However, a major obstacle in NP repair is the tissue's inherently limited self-regenerative capacity and progressive decline in physiological function. Given that NP tissue is characterized by its high hydration and gel-like consistency, which provides shock-absorbing properties, injectable gels have emerged as a promising therapeutic strategy for NP restoration and regeneration [20–23]. This strategy aims to alleviate IDD via a minimally invasive organohydrogel injection, which provides temporary mechanical support and bioactive cues for disc regeneration without requiring structural resection or fixation of the spine, thereby maintaining its intrinsic motion activity [24, 25].

To date, researchers have documented successful applications of hydrogels loaded with therapeutic drugs or cells for the treatment of IDD, with the purpose of creating a favorable microenvironment for NP regeneration. However, most injectable hydrogels used for NP repair undergo chemical crosslinking from a pre-gel state, which raises concerns regarding potential toxicity from cross-linking agents, insufficient gelling reactions and unpredictable changes in hydrogel characteristics and cargo behavior *in vivo* [26]. In addition, the mechanical properties of most reported hydrogels used for IDD treatment do not match the mechanical properties of natural NP tissues, nor can they provide continuous mechanical support in the IVD environment. Long-term overloading has been established as a key factor contributing to gradual NP tissue degradation and the failure of NP regeneration processes.

In recent years, polyvinyl alcohol (PVA) hydrogel has become a research hotspot in the treatment of intervertebral disc degeneration because of its similar mechanical properties to NP, but it can only provide mechanical support and lacks the biological activity to promote tissue repair [27–29]. To solve this problem, researchers added growth factors (such as TGF-β, GDF5) to PVA gel to activate the repair mechanism [30, 31]. However, growth factors are easy to be degraded by enzymes in the body, and conventional carriers are difficult to continuously release, resulting in a rapid decline in effective concentration and failure to achieve long-term repair. In a pioneering study by our group, a hydrogen bond crosslinked injectable organohydrogel (GPG) formulated by PVA and glycerol was reported, which could mechanically mimic native NP tissue in terms of load absorption and transfer postinjection [20]. This organohydrogel has no significant effect on nucleus pulposus cells (NPCs) under mechanical loading, and plays a certain role in repairing the IDD rat model. However, subsequent investigations revealed that this organohydrogel exhibited poor substrate adhesion, raising concerns about its potential migration under repeated mechanical overloading. Besides, despite its impressive water adsorption and retention ability, chemical stability and biocompatibility, the organohydrogel possesses insufficient elasticity, unfavored stiffness and limited bioactivity to induce NPCs generating extracellular matrix (ECM), which in consequence drives the investigation interest of PVA-based composite organohydrogel with additional modification.

Animal glue (AG) is a type of adhesive extracted from collagen proteins of mammals or fish [32, 33]. There are various types of AG on the market, such as animal skin and bone glue, fish maw glue, turtle shell glue and gelatin [34]. AG is mainly composed of collagen I, elastin, glycosaminoglycans and amino acids, and has been used as a wood adhesive and supplement in traditional Chinese medicine for centuries [35, 36]. Several groundbreaking studies have demonstrated the superiority of AG in tissue repair [37, 38]. For example, Xiao's team [39] has developed an AG based adhesive with excellent adhesion and minimal inflammatory response, suitable for wound healing and tissue engineering [40, 41]. However, it is worth noting that AG exhibits poor water resistance unless chemical crosslinking is applied. Considering the limited water content in NP and the exceptional adhesive and biological potential of AG, it is intriguing to integrate it into the developed GPG organohydrogel to develop a mechanical adaptive and tissue adhesive organohydrogel for NP repair.

Injectable hydrogels for intervertebral disc degeneration repair face critical challenges of insufficient bioactivity and poor mechanical compatibility. To address these limitations, this study developed a dynamically cross-linked GPG-AG organohydrogel through a ‘mechano-biological’ synergistic strategy. The system features a dual-network structure: a covalently cross-linked PVA/glycerol network provides fundamental mechanical stability, while glycerol's plasticizing effect enables dynamic network reconstruction to prevent brittle fracture typical of rigid materials. Simultaneously, AG forms complementary topological structures through physical entanglement with the PVA network, endowing the material with enhanced injectability and energy dissipation properties. The bioactive components in AG further promote tissue regeneration by regulating cellular behaviors. Notably, our facile preparation method utilizing commercially available AG cross-linked with the PVA/glycerol system yields a viscoelastic organohydrogel with dual functional advantages. This organohydrogel is expected to serve as an effective *in situ* platform for absorbing loads and maintaining the biological and structural integrity of degenerating NP.

## Materials and methods

### Organohydrogel preparation

Commercially available AG was purchased from Huizhou Qinjian Tools and Musical Instrument Accessories Co., Ltd., China. Conversely, PVA and glycerol were purchased from Sinopharm Chemical Reagent, China and Sigma-Aldrich, USA, respectively.

AG was soaked in deionized water at a 1:2 mass ratio, followed by exposure to a 65°C water bath with magnetic stirring to generate a viscous AG solution. Subsequently, a 5-min ultrasound treatment was applied to eliminate bubbles formed during the stirring process. Meanwhile, 1.2 g of PVA was dissolved in 18.8 ml of deionized water and stirred at 95°C to obtain a PVA solution. Then, 28.2 ml of glycerol was slowly added to create a well-mixed viscous GPG solution as described in our prior work [20]. Different volumes of the AG solution were added to the GPG solution (with GPG: AG volume ratios of 0.5:9.5, 1:9, 1.5:8.5 and 2:8), stirred at 65°C until a homogeneous PVA-glycerol-animal glue (GPG-AG) solution was achieved. All viscous solutions were later transferred to molds and allowed to cure at room temperature to obtain organohydrogels for further characterization. For comparison, GPG and AG organohydrogels were prepared via curing their precursor solutions and evaluated following the similar procedure of GPG-AG organohydrogel.

### Characterizations

#### Gelation behavior

The gelation time of GPG, AG and GPG-AG gels were measured at room temperature (RT) using the vial-inversion method, with the time recorded as the point when the sample could support its own weight. Furthermore, Fourier Transform Infrared Spectroscopy (FTIR) analysis was conducted to investigate the interactions between glycerol, PVA and AG during curing.

#### Compressive test

Cyclic compression tests were conducted on gel cylinder samples with a diameter of 15 mm and a height of 10 mm using a mechanical testing machine (CMT6104, China) operating at a speed of 10 mm/min. During these tests, the sample was compressed to a constant strain of 30%, and then, unloaded back to 0. Additionally, static compression tests were performed to determine the compression modulus of each specimen, which was calculated using the slope of the stress–strain curve at a strain of 10%.

#### Rheological test

A rheometer (AR2000, TA Instruments, USA) was used to evaluate the rheological properties of various formulated organohydrogels using a plate-to-plate setup. The parameters were set to 1% strain, with angular velocities ranging from 0.1 to 10 Hz at 37°C. The GPG-AG organohydrogel formula that exhibited the most NP tissue-like viscoelastic behavior was selected for further analysis.

#### Injection capability test

The injectability of prepared viscous solution was compared via measuring the force required to move the plunger to discharge the substance in syringe, known as dynamic slip force (DGF). Briefly, an uncured organohydrogel solution was loaded into a 10 ml syringe equipped with a 22 G needle and mounted onto the universal mechanical testing machine. The loading unit of the machine was set to a compression rate of 10 mm/min to simulate manual injection. The injection force during the process was recorded, and the DGF value was measured to assess the injection capacity of the solution.

#### Tissue adhesive test

An *ex vivo* sheep vertebrae model was utilized to investigate the distribution of organohydrogel solution postinjection, involving complete removal of the NP tissue and endplates. In test, uncured GPG, AG and GPG-AG gel solution with volume similar to the sucked NP was injected through the AF layer, and the distribution of the solution within the center of the IVD was visualized. Conversely, sample solution was injected to an intact intervertebral disc and exposed to 12 N load to check the leakage risk of injected organohydrogel visually.

#### Surface wettability, swelling capability and water retention capability

The wettability of the as-prepared organohydrogel was determined by measuring the contact angle equipment (CA, JC2000DM, China) in the sessile drop mode at room temperature. Using an automated micropipette, a 5-µl drop of Milli-Q water was carefully dispensed through a 0.7-mm inner diameter tip onto various randomly selected locations on the organohydrogel surface. The droplet's morphology was imaged, and the corresponding contact angle was recorded.

The relationship between the swelling capacity and the incubation time of the organohydrogel sample in water was established by measuring its free absorption capacity at regular intervals. The initial mass of the organohydrogel specimen in the wet state was noted as *W*_0_. The organohydrogel was then soaked in deionized water at room temperature and weighed every 60 min until it reached mass equilibrium. The final weight was recorded as *W*_s_, and the swelling rate could be calculated using the following equation:


$$\text{Swelling}\;\text{rate}=\left(W_{S}-W_{0}\right)/W_{0}\times 100\%.$$

The water retention capability of organohydrogel was tested via incubating specimens in chamber set at 37°C and 50% relative humidity. The initial weight of organohydrogel specimen was recorded as *W*_0_, and the weight at different time point was recorded as *W*_t_. The mass retention rate was calculated according to the formula below to compare water retention capacity:


$$\text{Mass}\;\text{retention}\;\text{rate}=W_{t}/W_{0}\times 100\%.$$

#### Degradation test

Organohydrogel specimen with weight recorded as W_0_ was incubated in a sealed tube containing PBS (pH = 7.4) at 37°C and its gradual mass change every day was tracked via measuring its weight (*W*_t_). The degradation rate of the organohydrogel could thereby be calculated as follows:


$$\text{Degradation}\;\text{rate}==W_{t}/W_{0}\times 100\%$$

### *In vitro* cellular test

#### Cell proliferation

In reference to ISO 10993-5, the cytocompatibility of organohydrogels was assessed by culturing rat NPCs (Wuhan Pricella Biotechnology Co., Ltd) using organohydrogel extracts for 1 and 3 days. The cells cultured with DMEM/F12 medium were set as the control group. Organohydrogel specimen was sterilized using 48 h ultraviolet light irradiation and incubated in 37°C DMEM/F12 cell culture medium at the ratio of 0.1 g/ml to prepare the extract. The as-prepared extract was then filtered using a 0.22 μm filter and mixed with 10% fetal bovine serum. NPCs were seeded into 96-well plates at a cell density of 5000/well and cultured for 24 h before exposing to extract. Subsequently, the cell culture medium was replaced with the extract and after 1 and 3 days the cell numbers were determined using CCK-8 kit. Meanwhile, in the control group cells were directly exposed to DMEM/F12 culture medium supplemented with 10% fetal bovine serum. Conversely, the viability of NPCs in the extracts for 24 h was assessed using live/dead staining and photographed using fluorescence microscopy. Additionally, the expression levels of ECM components such as collagen type II (Col II), aggrecan (ACAN) and MMP-13 were assessed using reverse transcription-polymerase chain reaction (RT-PCR) and immunofluorescence staining techniques.

#### Organohydrogel extract induced anti-inflammation regulation

Due to the persistent ischemic and hypoxic conditions within NP tissue during IDD, the equilibrium between the generation and elimination of reactive oxygen species (ROS) is severely perturbed. This event can gradually induce accumulation of substantial ROS cluster products *in situ*, activating downstream inflammatory signaling cascades. To simulate the oxidative stress microenvironment of the IVD, hydrogen peroxide (H_2_O_2_), a prototypical ROS, was selected. By employing staining techniques, we aimed to demonstrate the resistance of NPCs to this oxidative stress within the presence of organohydrogel extracts.

Prior to staining, a concentration of 100 μM H_2_O_2_ was introduced into the culture medium for 24 h to mimic the degenerative and inflammatory conditions within the IVD. A control group was maintained in normal culture medium without any oxidative stress induction. The experimental group involved incubating NPCs, pre-exposed to H_2_O_2_, with the organohydrogel extract for an additional 24 h. The inflammation protection capabilities of the organohydrogel were evaluated using ROS and Lipid peroxidation (LPO) staining methods. The test kits were purchased from Shanghai Beyotime Bio-chemical Technology Co., Ltd., In addition, the effect of organohydrogel extract on MMP13 expression in cells were also studied using fluorescence staining. In addition to imaging ROS level in cells using staining kit, fluorescence microplate reading method was also applied for ROS level evaluation. In brief, NPCs were cultured with different medium for 24 hours, digested with trypsin, and neutralized with serum-free medium, after which the cell suspension was transferred to centrifuge tubes and centrifuged to collect the cells; the cell pellet was resuspended in DCFH-DA working solution (10 μM, diluted in serum-free medium), incubated at 37°C in the dark for 30 minutes to ensure sufficient probe-cell contact, centrifuged again to collect the cells, washed 2-3 times with serum-free culture medium to completely remove extracellular DCFH-DA, resuspended in serum-free culture medium, seeded into a 96-well black microplate at 100 μl per well, and analyzed for intracellular ROS fluorescence intensity using a fluorescence microplate reader at an excitation wavelength of 500 nm and emission wavelength of 525 nm.

Conversely, the interaction of organohydrogel extract and macrophage cells was investigated. Briefly, RAW 264.7 macrophages (Chinese Academy of Sciences Cell Bank) were seeded into a 6-well plate and treated with lipopolysaccharide for 24 h to elicit cellular polarization. In the control group, they were maintained in normal culture medium in the absence of any stimulatory agent. In the experimental step, activated macrophages were incubated with organohydrogel extract for 24 h. Immunofluorescence staining techniques were employed to study the expression levels of both anti-inflammatory and pro-inflammatory cytokines. Furthermore, RT-PCR analysis was utilized to quantify the expression of the pro-inflammatory cytokine TNF-α and the anti-inflammatory cytokine IL-10 in testing groups.

### Animal study

All animal experiments conducted in this study have been approved by the Orthopaedic Animal Use and Care Committee of the First Affiliated Hospital of the Fourth Military Medical University of the Chinese People's Liberation Army (20220758). 20 male Sprague-Dawley rats aged 8 weeks were used in study. Following anesthesia administration, the skin of the rat tails was disinfected with alcohol and area of the C4-C5 intervertebral disc was marked using a marker pen. A 27-gauge needle was used to fully penetrate the AF layer, allowing for aspiration of the NP tissue. Complete removal of the NP tissue was indicated by the disappearance of sucking resistance. Subsequently, organohydrogel was injected into the center of NP.

Four weeks postsurgery, X-ray and CT examinations were performed on SD rats. The height of intervertebral discs in the normal group, puncture group, GPG group and GPG9-AG1 group were measured and compared, along with intervertebral disc height index (DHI) calculated.

### Data analysis

All data are expressed as mean ± standard deviation (*n* = 3). SPSS version 25.0 and Origin 2021 software were used for statistical analysis. A t-test was used for comparing two samples, while a one-way analysis of variance (ANOVA) was employed for comparing multiple samples. Statistically significant values are denoted as *P < 0.05, **P < 0.01 and ***P < 0.001, respectively.

## Results

### Organohydrogel preparation and characterizations

**Figure 1.**
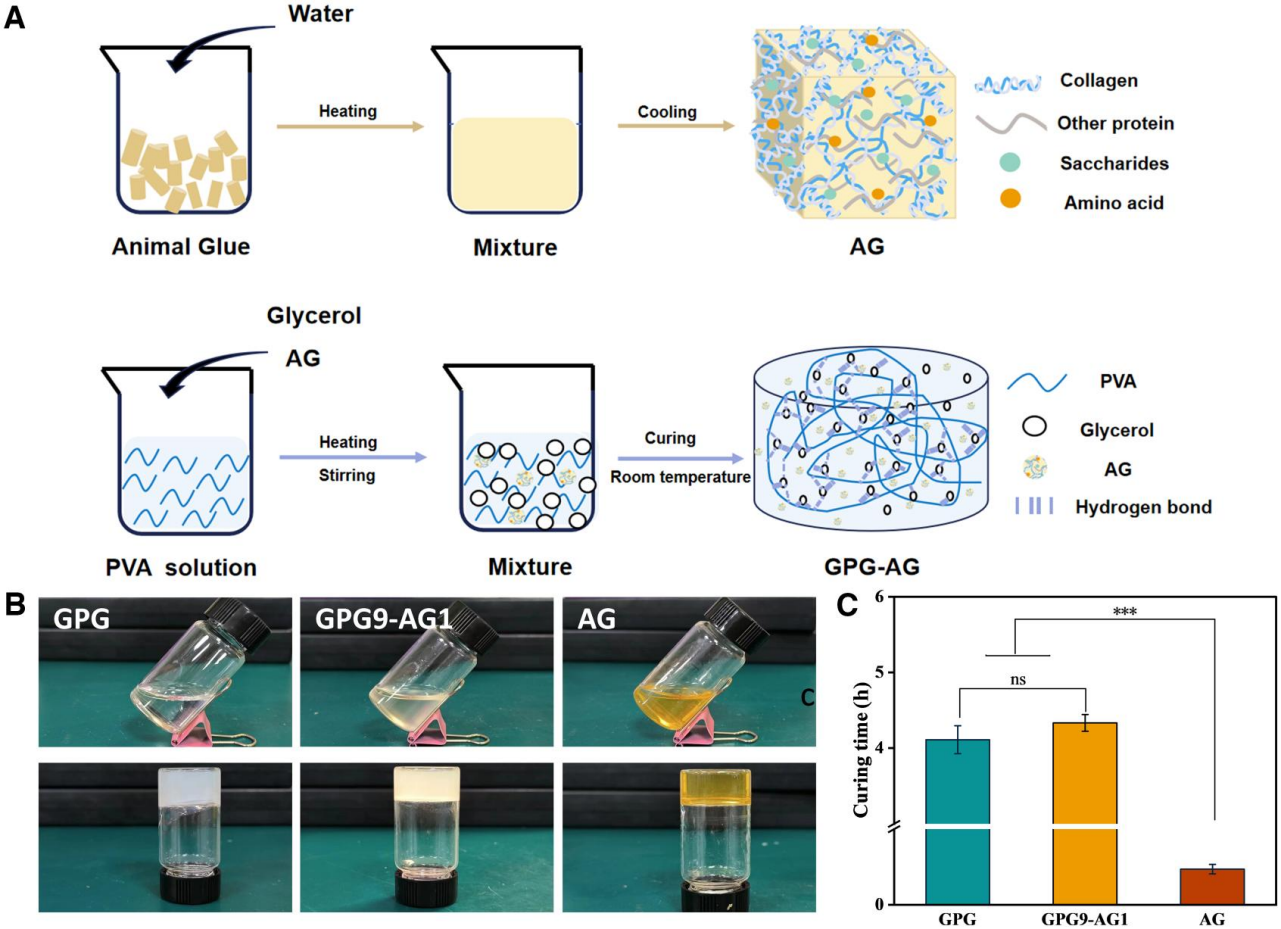
(**A**) Schematic diagram of preparation of AG and GPG-AG gels; (**B**) gels before curing (top) and after curing (bottom); and (**C**) curing time of GPG, GPG9-AG1 and AG. ***P < 0.001.

To preserve the load absorption capacity and structural integrity of compromised IVD, we developed a novel composite organohydrogel formed by previously reported GPG and an adhesive and bioactive AG. It was hypothesized that glycerol molecules could form multiple hydrogen bonds with both PVA and AG, leading to the formation of organohydrogel networks (Figure 1A). The gelation tests demonstrated that GPG, GPG9-AG1 and AG solutions could transit from a fluid-like state to a gel in the tube inversion test, with gelation times estimated to be 4.1 ± 0.19 h, 4.3 ± 0.1 h and 0.47 ± 0.06 h, respectively (Figure 1B and C). The fast gelation of AG was attributed to the high amount of gelatin derived from collagen during animal glue preparation. When exposed to room temperature, water molecules in the gelatin solution gradually become trapped within the gelatin network, forming a semi-rigid gel. Such gel-transition phenomena of organohydrogel enable their injection and temporary mechanical support in NP repairing practice.

**Figure 2.**
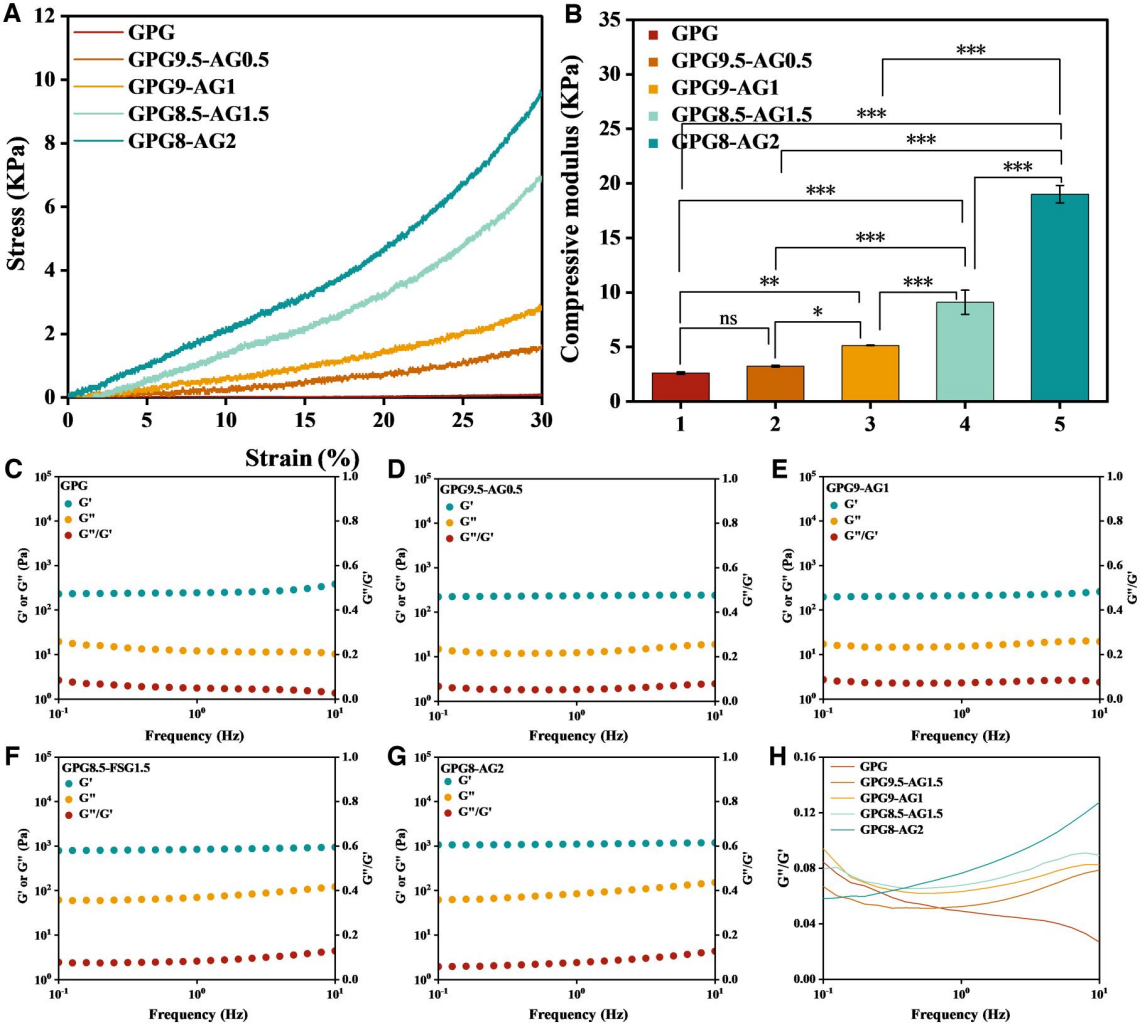
(**A**) Stress–strain curve of organohydrogel with different formula; (**B**) compressive modulus; (**C–G**) dependence of G′, G′′ and the G′′/G′ ratio (loss factor) of organohydrogel on the frequency of oscillation at 37°C and the strain of 1%; (**H**) loss coefficient. *P < 0.05, **P < 0.01 and ***P < 0.001.

A universal mechanical testing machine was used to conduct mechanical tests on the organohydrogel respectively at 30% compressive strain. It can be seen from Figure 2A that the higher AG content in GPG-AG, the higher stiffness could be witnessed. The slope of the first 10% strain segment in the stress-strain curve is defined as the compressive modulus of the sample, as shown in Figure 2B. Due to the superior elasticity of AG to GPG, the compressive elastic modulus of GPG-AG organohydrogel group showed an AG content dependent increase, in which the value is increased from 2.61 ± 0.11, 3.24 ± 0.09, 5.14 ± 0.02 kPa, 9.10 ± 1.10 kPa, to 19.01 ± 0.80 kPa, respectively. According to literature, the modulus of the human NP is 5.39 ± 2.56 kPa [42], close to the value of GPG9-AG1.

Conversely, the rheological properties of tested organohydrogels are depicted in Figure 2C–G, where it is evident that the energy storage modulus (G′) of all GPG-AG organohydrogels exceeded the energy dissipation modulus (G″), indicative of their viscoelastic solid state. As the concentration of AG increased, G′ exhibited an upward trend, attributed to the superior elasticity and stiffness of AG compared to GPG. Specifically, when subjected to a 1 Hz oscillation frequency, the GPG and GPG-AG organohydrogels exhibited energy storage modulus of 101.5 ± 0.81 Pa, 175.5 ± 6.7 Pa, 365.4 ± 1.68 Pa, 715.3 ± 17.4 Pa, 1139.7 ± 90.4 Pa, respectively. Notably, the G′ value of the organohydrogel with a GPG to AG ratio of 9.0:1.0 (365.4 Pa) approximated the G′ value of natural pig NP at 307 Pa [43]. Figure 2H shows the loss coefficient of organohydrogel. As the concentration of AG increases, the loss coefficient gradually increases, coupled with increasing viscosity and decreasing elasticity. Consequently, GPG9-AG1 organohydrogel comprising 90% GPG and 10% AG was chosen for further investigation.

**Figure 3.**
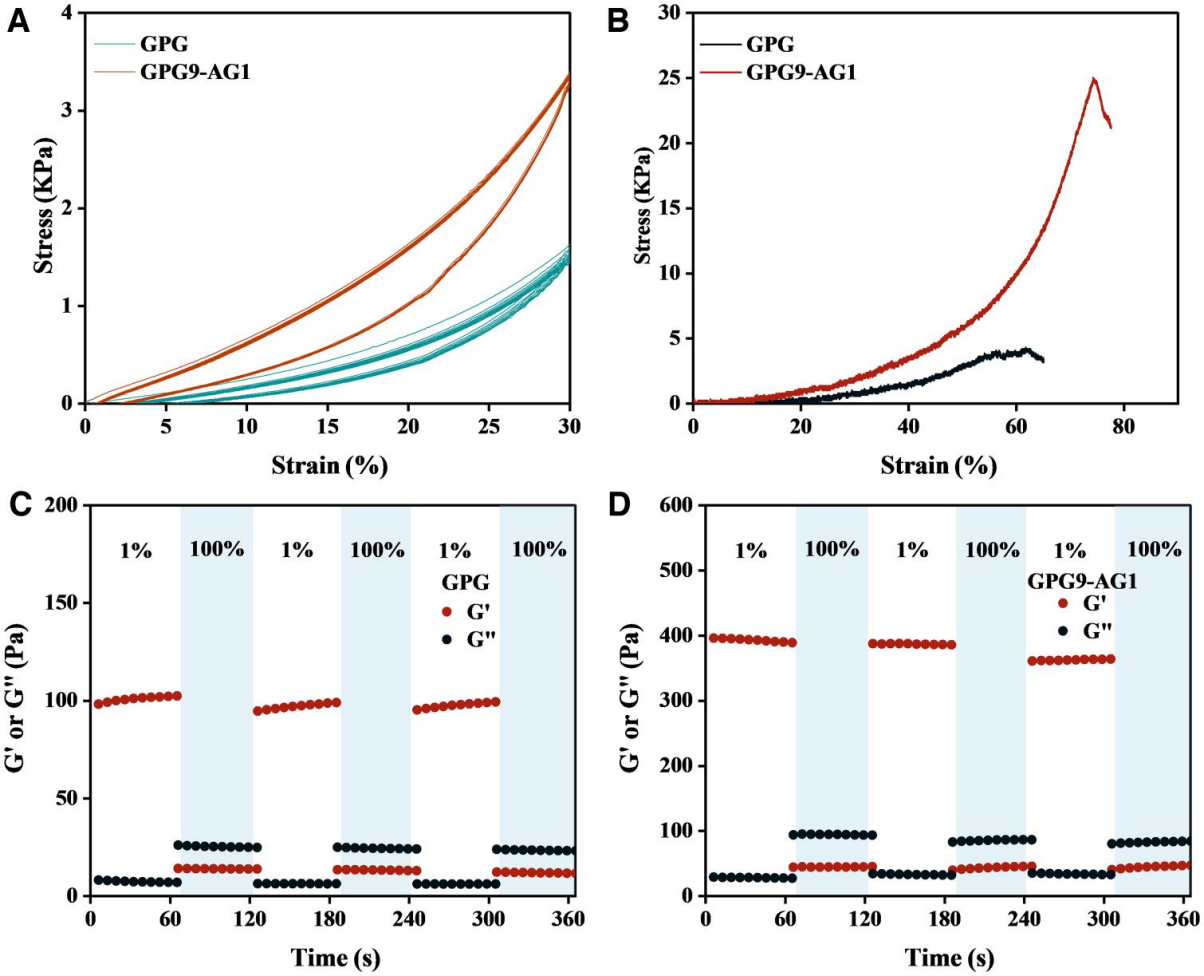
(**A**) Cyclic compressive stress–strain curve of GPG and GPG9-AG1; (**B**) ultimate strain; and time scanning of (**C**) GPG and (**D**) GPG9-AG1 under alternating strain of 100% and 1%.

Furthermore, the stress–strain curve of both GPG and GPG9-AG1 are shown in Figure 3A and B in which GPG breaks at about 62% strain, while GPG9-AG9 gel breaks at about 78% strain. These results indicated the latter one can withstand greater loads in practice. This can be explained by the greater mechanical performance of AG over GPG and the glycerol-mediated crosslinking could provide an energy-dissipating mechanism, both endowing energy absorbing capability to the as-formed GPG9-AG1 organohydrogel. The time scan results of GPG and GPG9-AG1 under alternating strain of 100% and 1% (Figure 3C and D) show that when they were at 1% strain, their G′ was greater than G″, indicating their viscoelastic solid state. When the strain increased to 100%, G′ dropd sharply and was lower than G′, indicating that the network structure of the organohydrogel was damaged. Finally, when the strain recovered to 1%, G′ and G″ immediately returned to their initial values, demonstrating their ability to heal rapidly. These observations also confirmed the superior reliability of GPG9-AG1 over GPG in restoring the mechanical environment of degenerated NP.

**Figure 4.**
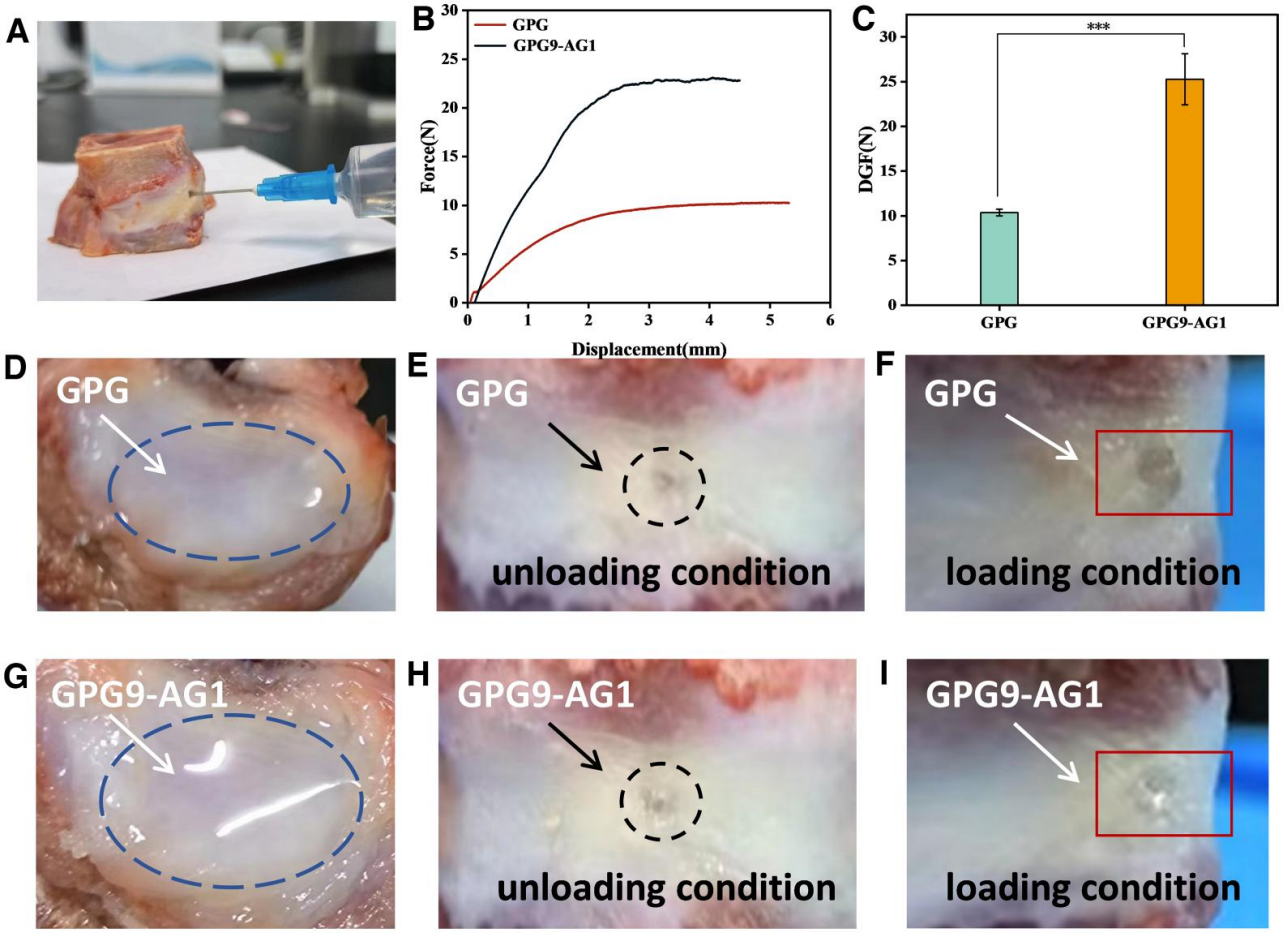
(**A**) Injection of organohydrogel *in vitro*; (**B**) injection force displacement curve of uncured injectable organohydrogel; (**C**) DGF of uncured injectable organohydrogel; (**D**) distribution of GPG in NP site postinjection; (**E**) injection hole of GPG in AF; (**F**) GPG leakage after loading; (**G**) distribution of GPG9-AG1 in NP site postinjection; (**H**) injection hole of GPG9-AG1 in AF; (**I**) no leakage of GPG9-AG1 after loading. ***P < 0.001.

Regarding NP located in the core of IVD, the injection capability of organohydrogel to NP was evaluated (Figure 4A). The injection forces of GPG and GPG9-AG1 in a 10 ml syringe equipped with a 22G needle are depicted in Figure 4B. Regarding the superior viscosity of GPG9-AG1 over GPG, more force was needed to carry out injection. The DGF during injection was estimated to be 10.37 ± 0.37 N and 25.28 ± 2.85 N, respectively (Figure 4C). Considering that an injection force of 40–50 N is generally convenient for most individuals [44], GPG9-AG1 can thereby smoothly injected by hand despite its higher viscosity to GPG. Conversely, both GPG and GPG9-AG1 were injected to NP site of ex vivo sheep vertebrae model. After 12 h of curing, both GPG and GPG9-AG1 were found to fully distributed in the NP location, as seen in Figure 4D and G. In addition, we found that under the unloaded condition after injection, as shown in Figure 4E and H, there was no leakage, while under the compressed postinjection, leakage of GPG fluid was witnessed at the injection hole on AF generated by needle punctuation (Figure 4F). In contrast, no leakage of GPG9-AG1 was found (Figure 4I), implying its superior adhesive capability to GPG. AG contains amino acids such as lysine (Lys) and arginine (Arg), which play an important role in the adhesion capability of materials by providing additional surface binding opportunities and repelling hydration layers. In addition, amino groups are believed to amplify cation-π interactions and enhance cohesive properties [45], playing a crucial role in protein and peptide self-assembly, molecular cohesion and adhesion and the formation of secondary structures [46, 47].

**Figure 5.**
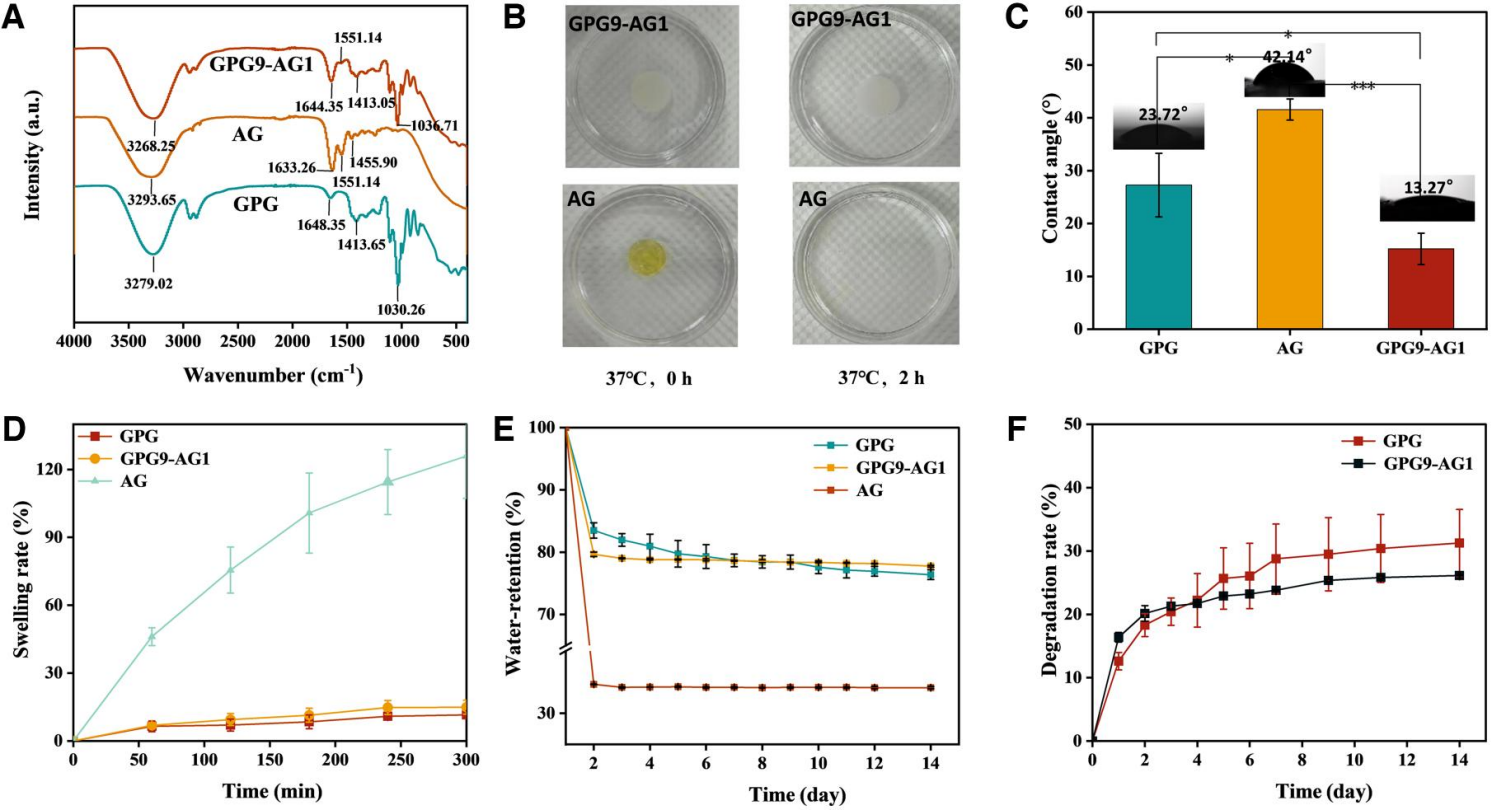
(**A**) Infrared spectrum of gel specimens; (**B**) structure integrity change of GPG9-AG1 and AG after incubation in 37°C water for 2 h; (**C**) contact angle; (**D**) swelling ability; (**E**) water retention rate; and (**F**) degradation rate of GPG9-AG1 and GPG. *P < 0.05 and ***P < 0.001.

The results of physical-chemical characterizations of GPG9-AG1 are demonstrated as follows: First, FTIR analysis was conducted to investigate the interaction between AG, glycerol and PVA (Figure 5A). The results revealed a shift in the -OH stretching peak and the C-O stretching vibration peak of AG in GPG9-AG1. Specifically, these peaks shifted from 3279.02 cm^−1–3268^.25 cm^−1^ and from 1030.26 cm^−1–1036^.71 cm^−1^, respectively [48–51]. Interestingly, in subsequent water incubation test, it was found cured AG show poor resistance to water invasion (Figure 5B). This behavior was caused by the weakening of interactions between molecular chains in solidified gelatin, which prevented the formation of a stable networked structure. On contrast, the GPG9-AG1 maintained its structure in water, possibly by the formation of hydrogen bonds (H-bonds) between glycerol molecules and collagen I, elastin and glycosaminoglycan found in AG.

Second, the wettability of GPG9-AG1 was assessed using the contact angle measurement method [52]. As illustrated in Figure 5C, the water contact angle of GPG9-AG1 (15.2 ± 2.97°) was significantly lower than that of pure AG (41.57 ± 1.99°) and GPG (27.27 ± 6.02°), suggesting its superior surface hydrophilicity. The improvement of hydrophilicity of organohydrogel is beneficial to cell adhesion and growth, and promotes tissue repair.

The results of the swelling test are summarized in Figure 5D. It was noteworthy that GPG exhibited limited water absorption capacity with a swelling rate of 1.5 ± 0.1%, potentially due to the crosslinked network formed between glycerol and PVA. As an alternative, AG demonstrated superior swelling ability (126 ± 18.7%), consistent with its poor water resistance addressed above. When AG was incorporated into GPG, the swelling rate increased to 14.9 ± 3.1%, as a comprehensive result of water affinity of AG and the formed crosslinked network among glycerol, PVA and AG. Additionally, the water retention rates of GPG9-AG1 and GPG were close when incubated at 37°C and 50% relative humidity for 14 days (Figure 5E). Conversely, the AG hydrogel lost 65.8% of its mass during the test. This outcome could be explained by the superior moisture retention capability of glycerol over glycosaminoglycan and collagen. The moisturizing properties of glycerol come from two aspects. On the one hand, the large number of hydrogen bonds formed between glycerol molecules and water molecules inhibit the evaporation of water; On the other hand, due to the hygroscopicity of glycerol itself, it can continuously capture water molecules from the surrounding environment. As the IVD degenerates, the ability of the NP tissue to absorb water decreases, leading to a decrease in IVD elasticity and deterioration of the microenvironment. The excellent water retention performance of glycerol can thereby restrict the water loss in NP to some extent. To further evaluate the degradation behavior of GPG9-AG1 and GPG, both organohydrogel specimens were incubated in PBS. As depicted in Figure 5F, after 14 days postimmersion, GPG exhibited a weight loss of 35%, while GPG9-AG 1experienced only a 21.6% weight loss. This difference could be caused by the hydrogen bond formation between glycerol and AG, enabling longer duration of GPG9-AG1 in NP to provide mechanical support and motivate tissue repairing.

### *In vitro* cellular test

**Figure 6.**
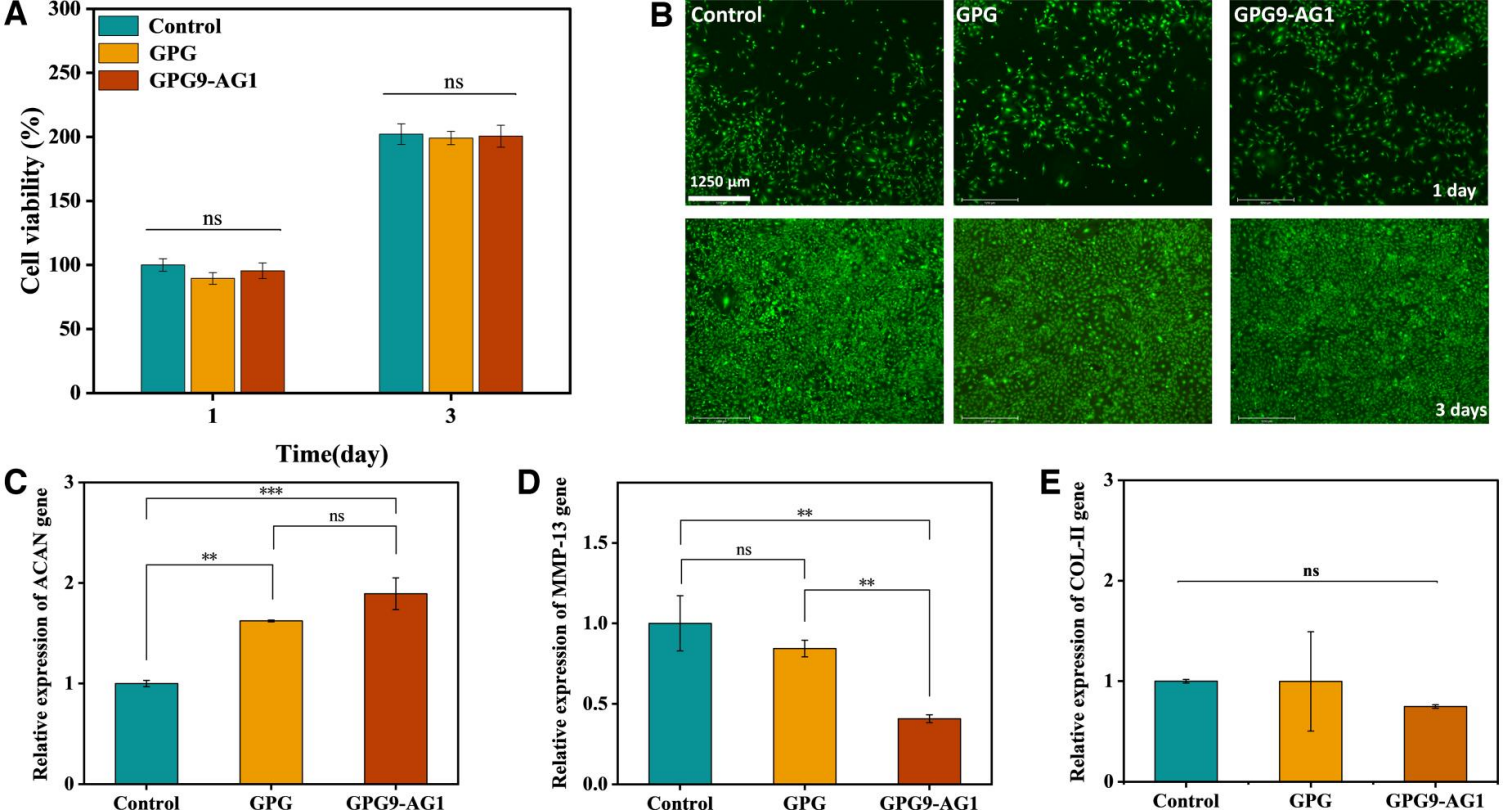
(**A**) CCK-8 of GPG and GPG9-AG1 extract after 1 and 3 days of culture with NPCs; (**B**) live/head staining photos of GPG and GPG9-AG1 extract with NPCs cultured for 1 and 3 days; (**C**) ACAN gene expression; (**D**) MMP13 gene expression; (**E**) COL-II gene expression. **P < 0.01 and ***P < 0.001.

As shown in Figure 6A, CCK-8 test method was used to evaluate the cell viability of GPG9-AG1 gel extract to verify its cytocompatibility. After incubating the NPCs with the organohydrogel extraction solution for 1 and 3 days, the cytotoxicity of organohydrogel specimens were compared. As shown in Figure 6A, no significant difference was found among the control, GPG and GPG9-AG1 group. The corresponding Live/Dead staining results shown in Figure 6B also confirmed the cytocompatibility of the GPG9-AG1 organohydrogel extract. The RT-PCR results (Figure 6C–E) revealed the expression of ACAN genes were greatly upregulated in GPG9-AG1 organohydrogel group along with the MMP-13 gene greatly downregulated. These observations indicate the GPG9-AG1 organohydrogel is cytocompatible and prone to accelerating ECM regeneration of NP.

**Figure 7.**
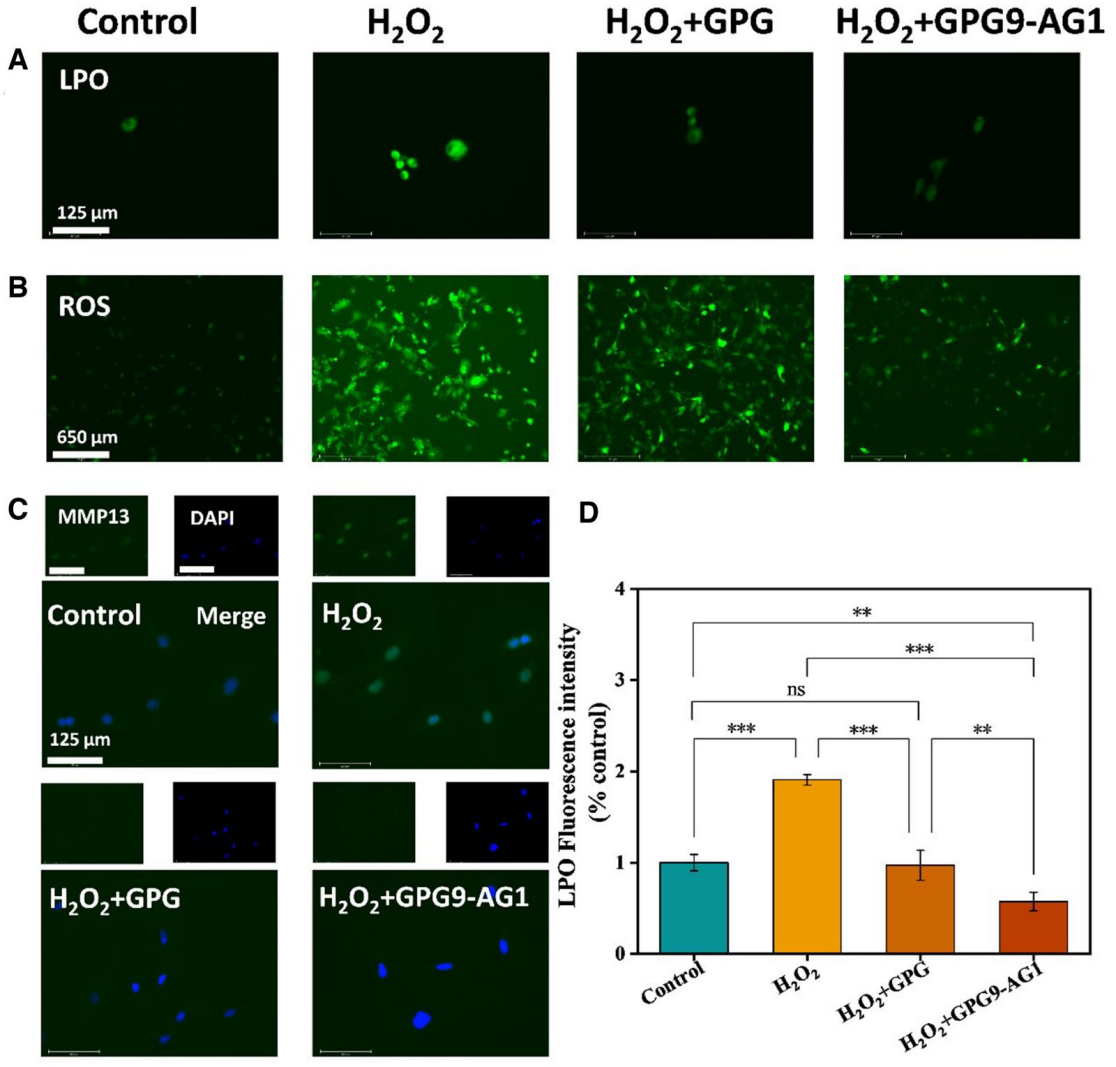
(**A**) LPO staining; (**B**) ROS staining; (**C**) MMP13 immunofluorescence staining; (**D**) LPO fluorescence intensity. *P < 0.05, **P < 0.01 and ***P < 0.001.

Regarding the pathological microenvironment in degenerated IVD, the equilibrium between the production and elimination of ROS is severely disrupted, stimulating downstream inflammatory pathways in consequence [53–56]. ROS induced LPO plays a critical role in cell death, characterized with disturbance of cell membrane organization and accompanied functional loss and modification of its proteins and DNA bases. The effect of organohydrogel extract on LPO protection was evaluated by applying a LPO fluorometric assay kit to H_2_O_2_ treated NPCs. The kit can provide a fluorescent probe that can detect LPO production in cells via reacting with the lipid free radicals in the LPO pathway. In test, the probe fluorescence green with the process of LPO, paving the possibility to measure the degree of LPO qualitatively and quantitatively. As shown in Figure 7A, compared with normal cells, H_2_O_2_ treated cells appeared significant green fluorescence intensity increase due to LPO level increase. However, when cells were exposed to both organohydrogel extract and H_2_O_2_, weakened green fluorescence was witnessed. Figure 7D shows the semi quantitative results of the fluorescence intensity of LPO. The outcomes implied GPG9-AG1 organohydrogel extract can protect cells from LPO significantly. On one hand, glycerol may be involved in cell protection against ROS via stabilizing the mitochondrial membrane potential and reducing intracellular ROS production [57, 58]. On the other hand, the AG can provide antioxidant in LPO restriction [59, 60].

Conversely, the level of ROS in H_2_O_2_ treated NPCs was also investigated. As shown in Figure 7B of representive ROS staining, the presence of GPG9-AG1 could significantly restrict the ROS related green fluorescence intensity. The similar results were witnessed using fluorescence microplate reading shown in Table 1. These observations were in agreement with the results found in LPO test. Besides, regarding MMP13 plays vital roles in degrading ECM and COL-II of NP, the impact of organohydrogel extract on MMP13 expression in NPCs were also studied. The immunofluorescence staining showed that GPG9-AG1 organohydrogel reduced the expression of MMP-13 in the presence of H_2_O_2_ (Figure 7C).

**Table 1.** Monitoring ROS production using fluorscence microplate reader

Group	ROS Fluorescence intensity (a.u.) (Ex/Em=500/525nm)								Mean ± SD
Control	884604	896229	883290	971134	982222	992115	996076	982385	948506.88±50752.49
H_2_O_2_	1279773	1237019	1228578	1195020	1106739	1093290	1093475	1097035	1166366.13±77052.62
H_2_O_2_+GPG	973791	982951	994364	1012567	1010418	1032448	1035049	1064955	1013317.88±30136.21
H_2_O_2_+GPG9-AG1	939144	915372	936341	924319	923230	912905	880763	938736	921351.25±19310.76

**Figure 8.**
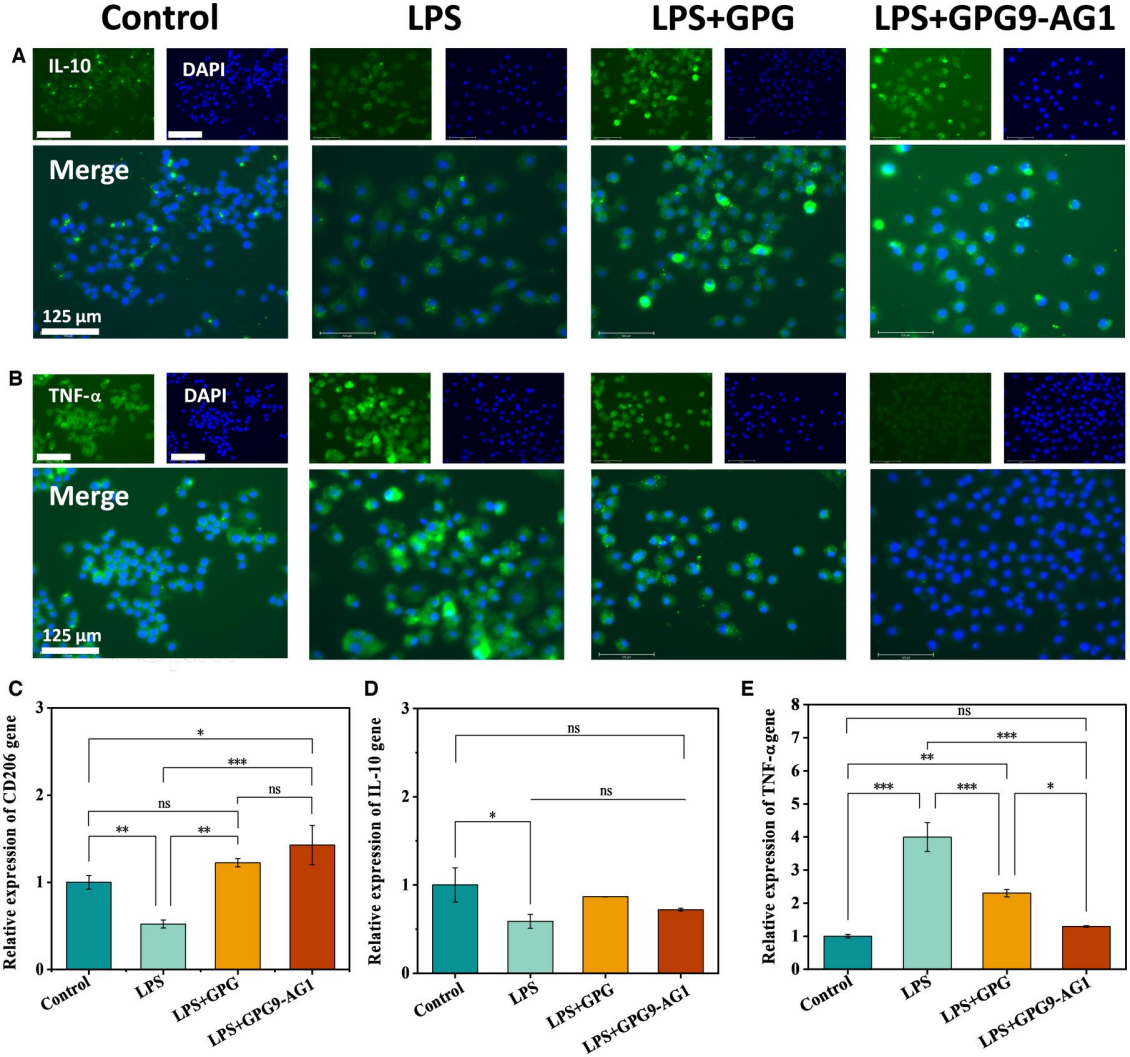
(**A**) IL-10 immunofluorescence staining; (**B**) TNF-α immunofluorescence staining; (**C**) CD206 gene expression; (**D**) IL-10 gene expression; and (**E**) TNF-α gene expression. *P < 0.05, **P < 0.01 and ***P < 0.001.

Next, macrophages were also used to investigate the anti-inflammatory potential of GPG9-AG1 organohydrogel extract. Typical anti-inflammatory cytokines such as interleukin (IL)-10 and pro-inflammatory cytokine TNF-α. As shown in Figure 8, LPS pre-activated macrophages were incubated with organohydrogel extract by immunofluorescence staining. Subsequently, immunofluorescence staining was used to detect the expression of IL-10 and TNF-α in different groups. The staining results showed that GPG9-AG1 organohydrogel extracts significantly inhibited TNF-α and promoted IL-10 compared with the control group without LPS stimulation (Figure 8A and B). The RT-PCR results (Figure 8C–E), GPG9-AG1 organohydrogel showed significant immunomodulatory and anti-inflammatory properties. The detection results of CD206, a marker for M2 macrophages, showed that the gene expression level in the LPS stimulated group was significantly reduced to 0.52 ± 0.05 times that of the control group, while the GPG based material group partially restored expression to 1.22 ± 0.05 times, but its promotion of M2 polarization was still significantly weaker than that of the GPG9-AG1 treated group at 1.43 ± 0.22 times (an increase of 175% compared to the LPS group). At the level of anti-inflammatory factor IL-10 expression, LPS stimulation significantly downregulated its gene expression level, while the GPG9-AG1 treatment group successfully reversed this trend and restored IL-10 expression to no statistically significant difference compared to the healthy control group. Regulation of the expression of pro-inflammatory cytokine TNF-α: LPS stimulation increased its expression level to 4.00 ± 0.44 times that of the normal group. Although GPG-based materials can partially inhibit the inflammatory response, adjusting it to 2.30 ± 0.11 times decreased it by 42.5% compared to the LPS group. However, the GPG9-AG1 treatment group showed superior anti-inflammatory efficacy, accurately regulating TNF-α expression to 1.29 ± 0.02 times, a decrease of 67.8% compared to the LPS group, and no significant difference from normal physiological status. RT-PCR further verified that GPG9-AG1 organohydrogel can effectively promote M2 phenotype polarization of macrophages and reduce inflammatory reaction. M2 macrophages play multiple important roles in the process of NP repair, including inhibiting inflammatory responses, promoting tissue repair and reconstruction and regulating immune responses [61]. They secrete anti-inflammatory cytokines such as IL-10 to suppress inflammatory responses, help alleviate tissue damage and repair damaged tissues [62]. In the late stage of inflammation, M2 macrophages can also inhibit the recruitment and activation of immune cells, thereby reducing further inflammatory responses [63–65]. In addition, M2 macrophages can promote fibrosis processes and help stabilize and repair damaged tissues. Therefore, the activation of M2 macrophages by GPG9-AG1 organohydrogel is suggested to alleviate IDD *in situ*.

### *In vivo* animal experiments

**Figure 9.**
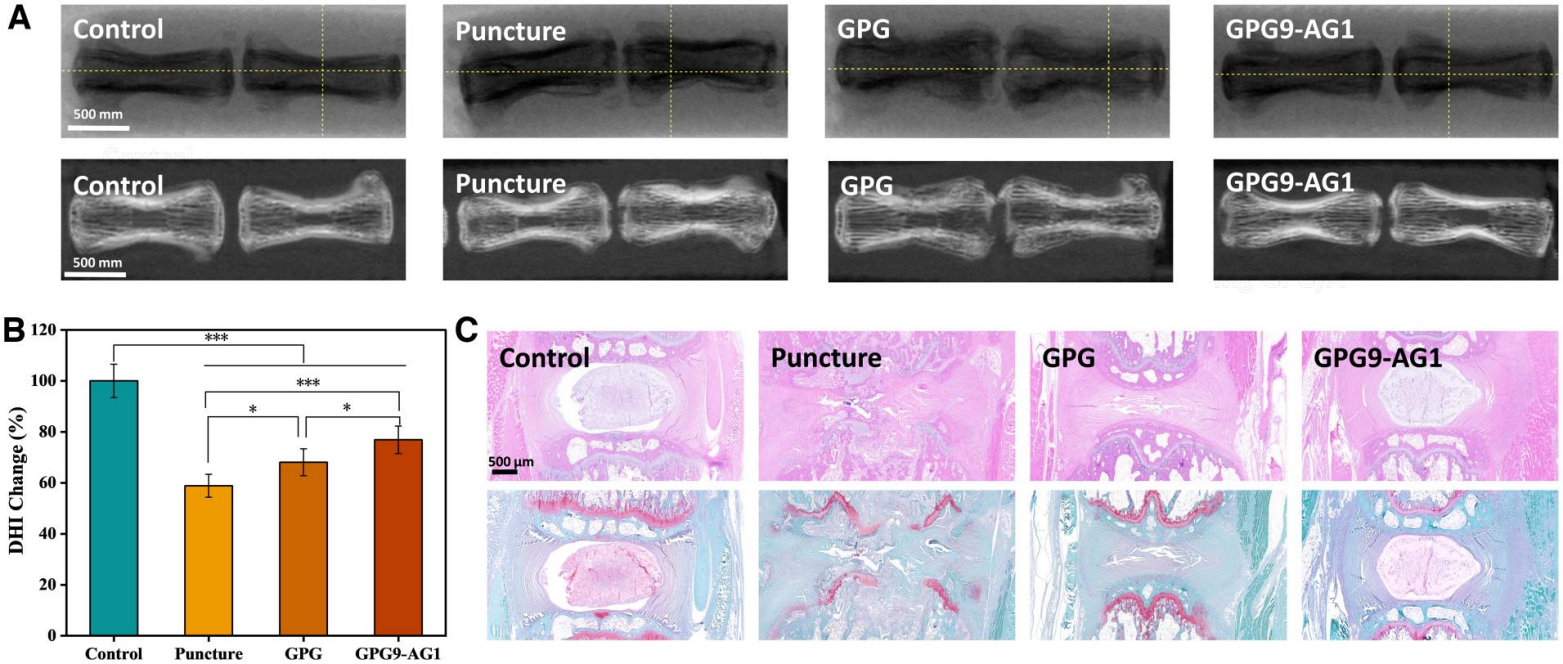
(**A**) X-ray, CT results of the intervertebral disc of rat tails at 4 w; (**B**) DHI% of the intervertebral disc of rat tails at 4 w; (**C**) hematoxylin & eosin staining (up) and Safranine-O/fast green staining (down) of the intervertebral disc of different groups at 4 w. *P < 0.05 and ***P < 0.001.

The change of intervertebral disc height index (DHI) is an important indicator for evaluating the level of IDD *in vivo*. Image J analysis software was used to measure the height of intervertebral discs and their adjacent vertebral bodies in the normal group, puncture group, GPG group, GPG9-AG1 group to calculate the DHI (Figure S6). The results showed that after 4 weeks postsurgery, the intervertebral disc height in the puncture group was reduced in the order of GPG9-AG1, GPG and puncture group, implying the as-fabricated organohydrogel as a certain therapeutic effect on IDD control (Figure 9A and B). Multidimensional morphological evaluation of intervertebral disc tissue was performed through H&E staining and Safranin O/Fast Green dual staining (Figure 9C). Experimental results indicated that GPG9-AG1 organohydrogel significantly promoted the repair of degenerated intervertebral discs. The normal control group displayed a typical hierarchical disc structure: the NP exhibited a plump elliptical morphology with uniformly distributed type II collagen networks, the annulus fibrosus showed densely arranged lamellar structures, the cartilaginous endplate line presented an intact undulating morphology and the subchondral bone structure remained undamaged. Compared to the normal group, the puncture injury group demonstrated characteristic degenerative changes: significant atrophy of the NP region, a drastic reduction in type II collagen content, multiple interlamellar fractures and torsional deformities in the annulus fibrosus, and disrupted continuity of the cartilaginous endplate line. The GPG control group showed partial improvement compared to the injury group, but the NP tissue was nearly absent, and the annulus fibrosus structure exhibited discontinuous alignment. The GPG9-AG1 treatment group revealed unique reparative characteristics: despite local compressive deformation in the annulus fibrosus caused by puncture manipulation, the NP region maintained near-normal cellular density, the extracellular matrix displayed a homogeneous three-dimensional reticular distribution of type II collagen expression, the endplate structure remained intact with a continuous undulating cartilaginous line, and the degree of degeneration was lower than that in both the injury group and GPG group.

## Discussion

The rheological test results show that GPG9-AG1 has solid gel properties, and the G′ and G′′ of GPG9-AG1 are comparable to the natural NP. In addition, due to the dynamic fracture/restoration of hydrogen bond generated by PVA and glycerol, organohydrogel can withstand the mechanical cycle compression and show effective energy dissipation, with a certain degree of self-healing GPG-AG can adhere to the disc tissue to prevent leakage. GPG9-AG1 gel has good hydrophilicity and water retention, and can maintain water. *In vitro* cell experiments have shown that GPG9-AG1 has good biocompatibility, inhibits lipid peroxidation, and clears ROS. In addition, it can promote the secretion of anti-inflammatory factors and improve the microenvironment of the NP. Animal experiments show that GPG-AG organohydrogel significantly restores the height of intervertebral disc, promotes the repair of NP and effectively inhibits the degeneration of intervertebral disc.

The GPG-AG organohydrogel exhibits multidimensional advantages over chitosan-based or hyaluronic acid injectable gels [66, 67]. Its dynamically reversible hydrogen-bonded network endows the hydrogel with viscoelastic properties that closely match those of native NP tissue and exceptional fatigue resistance, effectively avoiding the stress-shielding effects common in traditional gels. In terms of biocompatibility, GPG-AG induces M2 macrophage polarization, promotes NPCs proliferation and facilitates matrix nutrient exchange, whereas materials like chitosan may exacerbate inflammatory responses. Therapeutically, the hydrogel alleviates annular fibrosus stress by restoring intervertebral disc pressure, synergistically upregulates Aggrecan expression and drives the regeneration of type II collagen and proteoglycans, significantly outperforming purely mechanical-support gels [28, 68]. By overcoming the long-standing trade-off between mechanical support and bioactivity in conventional materials, GPG-AG provides an innovative repair strategy for disc degeneration that integrates biomechanical compatibility, immunomodulation and matrix regeneration.

The degradation kinetics of GPG-AG synergize with the NP repair process. Its dynamic hydrogen-bonded network enables time-dependent regulation of degradation rates through controlled dissociation, ensuring mechanical support in early stages and space release in later phases to guide endogenous regeneration. Meanwhile, degradation products modulate macrophage polarization to suppress fibrotic pathways and activate NP progenitor cell migration/differentiation, forming a ‘degradation-immunomodulation-regeneration’ positive feedback loop. This time-dependent functional transition achieves dynamic mechanical adaptation and bioactive synergy during degradation, ultimately providing progressive structural-functional restoration for degenerated discs.

The GPG-AG organohydrogel developed in this study shows significant advantages in terms of mechanical adaptability, biological activity and repair effect of intervertebral disc degeneration, but there are still the following limitations that need further discussion:

The thermosensitivity of AG poses a challenge of operational adaptability in clinical application. Its sol gel transformation needs to rely on special preheating equipment (such as constant temperature water bath) and extend the preparation time during operation. To improve clinical operability, immediate gelation during surgery can be achieved through material chemical modification strategies such as photoresponsive group modification (such as methacrylation) or enzyme triggered crosslinking (such as transglutaminase mediated), or through dosage form innovation (such as developing a freeze-dried powder liquid two-component instant mixing system), thereby avoiding the need for heat pretreatment.

Although rat NPC experiments have shown that GPG-AG can inhibit lipid peroxidation, clear ROS and promote the secretion of anti-inflammatory factors, there are species differences between rat and human NPCs in matrix metabolism (such as proteoglycan/type II collagen synthesis rate) and inflammatory regulatory pathways, which may affect the accuracy of efficacy prediction. Subsequently, its biological effects need to be validated by combining human NPCs or degenerative intervertebral disc organoid models.

## Conclusions

Injectable organohydrogel (GPG9-AG1) was prepared by a simple one pot method using PVA, glycerin and AG. The compression modulus of GPG9-AG1 is 5.14 ± 0.02 kPa, which is close to that of natural NP and can withstand about 78% of strain. The storage modulus of GPG9-AG1 organic gel is found to be 365.4 ± 1.68 Pa, equivalent to natural NP as well. Moreover, due to the dynamic fracture/restoration of hydrogen bonds between PVA, AG and glycerol, it can withstand cyclic loads and exhibit effective energy dissipation capabilities. *In vitro* studies have shown that GPG9-AG1 is non-toxic to NPCs and promote ACAN gene expression. In addition, this organohydrogel enabled ROS and LPO alleviation, MMP-13 inhibition and activate M2 macrophages activation, paving the possibility to regulate a favored microenvironment for NP tissue regeneration. *In vivo* studies have shown that GPG9-AG1 could maintain intervertebral disc height to some extent in NPD models as compared to GPG and blank control. The results indicate that GPG9-AG1 has great potential for transformation in the minimally invasive treatment of IVDD. GPG-AG organohydrogel shows an important clinical application prospect in the minimally invasive treatment of intervertebral disc degeneration, providing a dual mechanical biological repair function of mechanical adaptation and microenvironment regulation for degenerative intervertebral discs. To further promote transformation, it is necessary to focus on analyzing the key molecular mechanisms that regulate the inflammatory microenvironment, such as revealing its targets for inhibiting inflammatory responses and promoting NP repair through multi-omics techniques. In the process of promoting clinical translation, it is necessary to construct a long-term evaluation model for non-human primates (implantation period ≥ 18 months), systematically evaluate the *in vivo* metabolic trajectory of material degradation products, dynamic changes in local immune microenvironment and the long-term maintenance effect of intervertebral disc biomechanical function.

## Supplementary Material

rbaf047_Supplementary_Data

## Data Availability

Data will be made available on request.
