# Supplementary material for: Development of a mechanical adaptable, moisture retention capable, injectable and adhesive organohydrogel for nucleus pulposus repairing
Source: Regen Biomater. 2025 May 19;12:rbaf047. doi: 10.1093/rb/rbaf047 (PMC12202143; doi:10.1093/rb/rbaf047)
Supplement: rbaf047_Supplementary_Data [file rbaf047_supplementary_data.zip › Support Information.docx]

**Support Information**

**Development of a mechanical adaptable, moisture retention capable, injectable, and adhesive organohydrogel for nucleus pulposus repairing**

Yaping Wang^a^, Dong Wang^b^, Chu Gao^b,c^, Chouxin Zhou^b^, Xiao Lin^d^, Di Wang^b^, Liu Yang^b,c,*^, Huan Zhou^a,*^, Lei Yang^a,*^

^a^ Center for Health Science and Engineering, Hebei Key Laboratory of Biomaterials and Smart Theranostics, School of Health Sciences and Biomedical Engineering, Hebei University of Technology, Tianjin, 300131, China

^b^ Institute of Orthopedic Surgery, Xijing Hospital, Fourth Military Medical University, Xi’an, People’s Republic of China

^c^ Medical Research Institute, Northwestern Polytechnical University, Xi’an, People’s Republic of China

^d^ Orthopedic Institute and Department of Orthopedics, The First Affiliated Hospital, Soochow University, Suzhou, 215000, China

* Corresponding author. E-mail addresses: yangliu@fmmu.edu.cn (L. Yang), zhouhuan@hebut.edu.cn (H. Zhou), ylei@hebut.edu.cn (L. Yang)

**METHODS AND MATERIALS**

1. **Preparation and evaluation of GPG-AG organohydrogel**

**1.1. Preparation of GPG-AG organohydrogel**

PVA: average molecular weight: 22000, polymerization degree 1750, Sinopharm Chemical Reagent Co, Ltd.,Shanghai, China .Glycerol:reagent grade, 99%, Sigma-Aldrich

Dissolve 1.2g of PVA in 18.8mL of deionized water and stir at 95 ℃ to obtain a PVA solution. Then, as described in our previous work , slowly add 28.2 mL of glycerol to form a well mixed viscous GPG solution. Add AG solutions of different volumes to GPG solution and stir at 65℃ until a uniform PVA glycerol animal gel (GPG-AG) solution is obtained. All viscous solutions were then transferred to the mold and cured at room temperature to obtain an organohydrogel for further characterization. For comparison, GPG and AG organohydrogel were prepared by curing their precursor solutions and evaluated according to similar procedures for GPG-AG organohydrogel.The ratio of GPG-AG organohydrogels is shown in Table SI.

**1.2. Mechanical property of GPG-AG organohydrogel**

GPG-AG organohydrogels was made into cylindrical samples with a diameter of 15 mm and a height of 10 mm. After they were placed in 37 ℃, -4 ℃ and -20 ℃ for 24 hours, the samples were compressed to 30% of the constant strain to measure their mechanical properties. Prepare cylindrical samples again, place them in buffer solutions with pH=5.5 and pH=7.4 for 24 hours, and then compress the samples to a constant strain of 30% to measure their mechanical properties. Each group consists of 3 samples.

**1.3. Swelling property of natural nucleus pulposus**

Extract the nucleus pulposus tissue from the ex vivo sheep spine model, with an initial mass of W_0_. Then immerse the nucleus pulposus in deionized water at room temperature and weigh it every 60 minutes until mass balance is reached. The final weight is denoted as W_S_, and the swelling rate can be calculated using the following equation:

Swelling rate=(W_S_-W_0_)/ W_0_×100%

**1.4. Injection force of natural nucleus pulposus**

Put the uncured organohydrogel solution into syringes with 21G, 22G, 27G and 30G needles respectively, and install them on the universal mechanical testing machine. Set the loading unit of the machine to a compression rate of 10mm/min to simulate manual injection. Record the injection force during the process to evaluate the injection ability of the solution.

**2.Quantitative real-time PCR analysis**

NPCs are placed in 6-well tissue culture plates at a density of 10 ^ 5/ml. After 24 hours, incubate NPC with organohydrogel extract. After 3 days of incubation, extract total RNA. According to the manufacturer's requirements, RNA was extracted from cells using an RNA extraction kit (Biyun Tian, China). CDNA was synthesized using the primescript RT kit (Biyuntian, China), and after reverse transcription reaction, RT PCR was performed using the SYBR Green qRT PCR kit (Biyuntian, China) and a real-time PCR system (Bole Life Medical Products Co., Ltd.). Three copies of Gapdh are provided for each sample as a reference. Table S2 provides the sequences of the primers used.

**3.Radiology evaluation**

Measure the intervertebral disc height and adjacent vertebral body height of the normal group, acupuncture group, GPG group, and GPG-AG group using Image J software, and calculate the intervertebral disc height index (DHI). Subsequently, the DHI of the GPG group, GPG-AG group, and acupuncture group were compared with the DHI of the normal group, and the DHI percentage (DHI%) was calculated to evaluate the degree of degeneration of intervertebral discs in each group at different time points compared to the normal group ( Figure S6).

**SUPPLEMENTARY FIGURES**

**Figure S1. Mechanical properties of organohydrogels at different temperatures.**

**Figure S2. Mechanical properties of organohydrogels at different pH.**


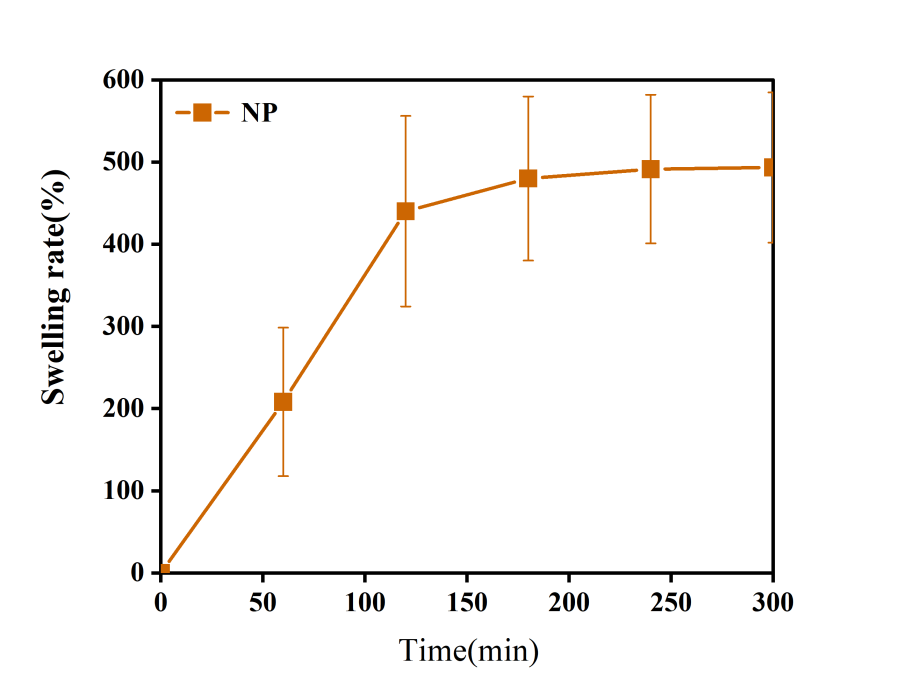


**Figure S3. Swelling properties of natural nucleus .**


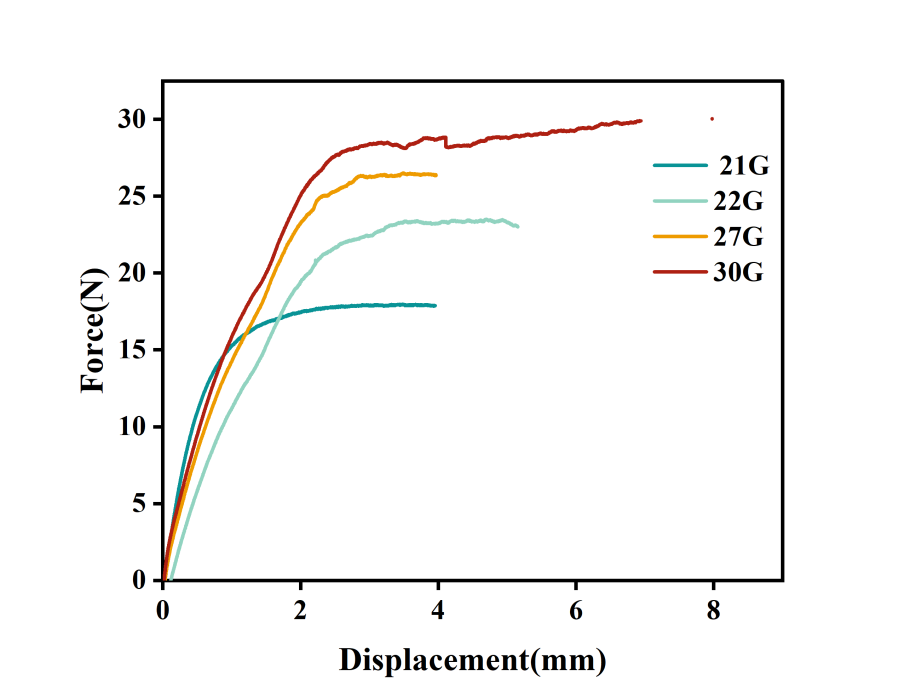


**Figure S4. Evaluation of injectability of organohydrogels**

**with different types of needles .**


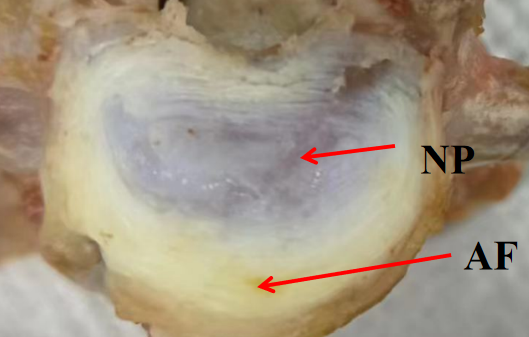


**Figure S5. NP and AF in ex vivo model**


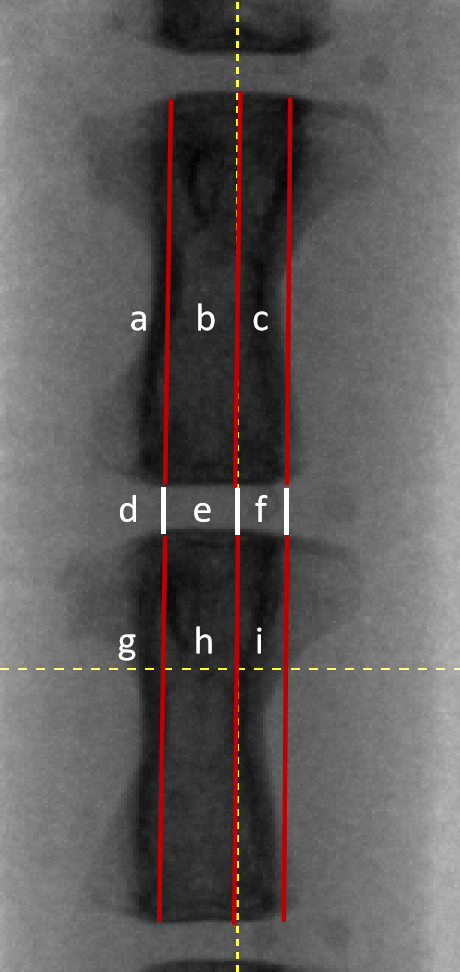


**

**Figure S6. DHI% was measured from digitized radiographs**

**SUPPLEMENTARY TABLES**

Table S1 Formulation of GPG-AG organohydrogels

| Group | GPG/ml | AG/ml |
| --- | --- | --- |
| GPG | 10 | 0 |
| GPG9.5-AG0.5 | 9.5 | 0.5 |
| GPG9-AG1 | 9 | 1 |
| GPG8.5-AG1.5 | 8.5 | 1.5 |
| GPG8-AG2 | 8 | 2 |
| AG | 0 | 10 |

Table S2 qRT-PCR primers

| Gene(Rattus norvegicus) | | Sequence(5’- 3’) | |
| --- | --- | --- | --- |
| Col II | Forward primer | | ATAGCAAATCGGCTGACGGT |
|  | Reverse primer | | CGCTCTCACCCTTCACACCT |
| Acan | Forward primer | | AGGATGGCTTCCACCAGTGC |
|  | Reverse primer | | TGCGTAAAAGACCTCACCCTCC |
| MMP-13 | Forward primer | | GCAGCTCCAAAGGCTACAA |
|  | Reverse primer | | CATCATCTGGGAGCATGAAA |
| Gapdh | Forward primer | | AGTGCCAGCCTCGTCTCATA |
|  | Reverse primer | | GACTGTGCCGTTGAACTTGC |
| CD206 | Forward primer | | GGAGTGGCAGGTGGCTTATG |
|  | Reverse primer | | CACTGCTCGTAATCAGCCTCC |
| IL-10 | Forward primer | | AATAAGCTCCAAGACCAAGGTGT |
|  | Reverse primer | | CATCATGTATGCTTCTATGCAGTTG |
| TNF-α | Forward primer | | ACCCTCACACTCACAAACCA |
|  | Reverse primer | | ATAGCAAATCGGCTGACGGT |
