# Supplementary material for: Development of a mechanical adaptable, moisture retention capable, injectable and adhesive organohydrogel for nucleus pulposus repairing
Source: Regen Biomater. 2025 May 19;12:rbaf047. doi: 10.1093/rb/rbaf047 (PMC12202143; doi:10.1093/rb/rbaf047)
Supplement: rbaf047_Supplementary_Data [file rbaf047_supplementary_data.zip › 4.28.docx]

Dear Editor and Reviewers,

Many thanks to your patient review, helpful comments and kind encouragement. We have carefully revised our manuscript according to the given comments and suggestions. In our revised manuscript, the results and discussion were polished, along with supplemental results requested by reviewers. The point-to-point response and revision were carefully addressed as attached.

Many thanks again for your consideration and suggestions!

Best regards,

Huan Zhou, on behalf of all authors

**Referee: 3**

1. Abbreviation Consistency

- After defining an abbreviation (e.g., Nucleus Pulposus as NP), the abbreviated form (NP) should be used consistently throughout the manuscript. Currently, nucleus pulposus appears over 40 times in complete form, affecting conciseness. Revising this will improve readability.

Response: Thank you for highlighting this important issue. We have systematically replaced all subsequent mentions of "Nucleus Pulposus" with the abbreviation "NP" after its initial definition in the Introduction section. This revision has improved the manuscript's conciseness while maintaining scientific clarity.

2. Journal-Specific Abbreviation Rules

- As stated in the journal’s guidelines under "Abbreviations", non-standard abbreviations must be defined at first use and should only be introduced if the term appears multiple times. Additionally, abbreviations should not be used in headings or keywords. Specific issues include:

- "ROS" (Reactive Oxygen Species) is used in the abstract but not defined upon first occurrence. Please explain it when it is introduced.

- The keyword list may need revision if abbreviations are currently included. Consider using the complete form of the term instead of the abbreviation to improve searchability and understanding for readers.

Response: We sincerely appreciate the reviewer’s attention to terminology rigor. In response to your comment:

Definition added in the abstract: The term "Reactive oxygen species (ROS)" is now explicitly defined upon its first occurrence.

Keyword adjustment: The keyword list has been revised to include the full term "Reactive oxygen species" instead of the abbreviation "ROS", "Intervertebral disc degeneration" instead of the abbreviation "IDD", aligning with journal guidelines.

3. Punctuation Errors

- Some punctuation issues remain, such as missing periods (e.g., in the Introduction: “… acceleration[5][6] Physiologically, …”). Please ensure proper punctuation throughout the manuscript.

Response: Thank you for your meticulous feedback. We have thoroughly revised punctuation throughout the manuscript, including adding missing periods (e.g., in the Introduction: “… acceleration[5][6] Physiologically, …” to “along with increasing prevalence as global population aging acceleration[6][7]. Physiologically, …” in the Introduction). All punctuation now adheres to academic writing standards.
